# Successful Abdominal Section for Intussusception

**Published:** 1874-04

**Authors:** 


A Successful Case of Abdominal Section for Intussusception was reported by Mr. Hutchinson at a late meeting of the Royal Medical and Chirurgical Society. He appended to the report the following observations:—

1. That it is by no means very uncommon for intussusception to begin at the ilio-cæcal valve, and to progress to such a length that the invaginated part is within reach from the anal orifice, or even extruded.

2. That it is of great importance in all cases of suspected intussusception to examine carefully by the anus.

3. That in almost all cases of intussusception in children, and probably in most of those in adults, the diagnosis may be made certain by handling the invaginated part through the abdominal wall.

4. That the prognosis of cases of intussusception varies much; first, in ratio with the age of the patient, and, secondly, with the tightness of the constriction.

5. That in a large proportion of the cases in which children under one year are the patients, death must be expected within from one to four or six days from the commencement.

6. That in the fatal cases death is usually caused by shock, or by collapse from irritation, and not by peritonitis.

7. That in many cases it is easy, by estimating the severity of the symptoms (vomiting, constipation, &c.,) to form an opinion as to whether the intestine is strangulated or simply irreducible.

8. That in cases of strangulated intussusception, whilst that is great risk of speedy death, there is, also, some hope that grangrene may be produced, and spontaneous cure result.

9. That in cases in which the intussuscepted part is incarcerated and not strangulated, there is very little hope of the occurrence of grangrene, and it is probable that the patient will, after some weeks or months, die, worn out by irritation and pain.

10. That the chances of successful treatment, whether by the use of bougies, or by the injection of air or water, are exceedingly small, excepting in quite recent cases, and that if the surgeon does not succeed by them promptly, it is not likely that he will succeed at all.

11. That the cases best suited for operation are those which have persisted for some considerable time, and in which the intestine is only incarcerated; and that these cases are also precisely those least likely to be relieved by any other method.

12. That in the cases just referred to, after failure by injections, bougies, &c., an operation is to be strongly recommended.

13. That the records of *post-mortems* justify the belief that, in a considerable number of the cases referred to, the surgeons will encounter no material difficulty after opening the abdomen.

14. That the circumstances which might cause difficulty are—(1) the tightness of the impaction of the parts; (2) the existence of adhesions; and (3) the presence of gangrene.

15. That, in selecting cases suitable for operation, the surgeon should be guided by the severity of the symptoms to an estimate of the tightness of the strangulation, and as to the probability of gangrene already set in.

16. That, in cases in which the patient's symptoms are very severe, or the stage greatly advanced, it may be wiser to decline the operation, and trust in the use of opiates.

17. That the operation is best performed by an incision in the median line below the umbilicus.

18. That, in cases of intussusception in young infants (under one year of age,) the prognosis is very desperate, scarcely any recovering, excepting the few in whom injection treatment is immediately successful, whilst a large majority die very quickly.

19. That the fact just mentioned may be held to justify, in the case of young infants, very early resort to the operation.

20. That it is very desirable that all who, in the future, have the opportunity for *post-mortem* examination of intussusception cases should give attention to the question as to whether an operation would have been practicable, and should record their results.—*The Doctor.—Boston Med. and Surg. Jour.*

